# Supplementary material for: The Effect of Low Doses of Zearalenone (ZEN) on the Bone Marrow Microenvironment and Haematological Parameters of Blood Plasma in Pre-Pubertal Gilts
Source: Toxins (Basel). 2022 Jan 29;14(2):105. doi: 10.3390/toxins14020105 (PMC8880195; doi:10.3390/toxins14020105)
Supplement: Supplementary file 1 [file toxins-14-00105-s001.zip › toxins-1571387-supplementary.pdf]

toxins

MDPI

Supplementary Materials

The Effect of Low Doses of Zearalenone (ZEN) on the Bone Marrow Microenvironment and Haematological Parameters of Blood Plasma in Pre-Pubertal Gilts

Magdalena Mróz, Magdalena Gajęcka, Katarzyna E. Przybyłowicz, Tomasz Sawicki, Sylwia Lisieska-Żołnierczyk Łukasz Zielonk and Maciej Tadeusz Gajęcki

Table S1. Selected haematological parameters in group C on different analytical dates ($\bar{x}$, SD).

Blood collection dates	WBC 10^9/L	NEUT %	LYMPH %	RBC 10^{12}/L	HGB g/L	HCT %	MCHC g/dl	RDW %	HDW g/dl
1	23.74 ± 0.39 ^{ddg}	45.06 ± 7.33	47 ± 6.71	6.49 ± 0.67 ^{efg}	10.16 ± 0.54	35.48 ± 2.45 ^{eefgg}	28.72 ± 0.71^{e}	17.62 ± 1.46	1.61 ± 0.17 ^{d}
2	20.59 ± 5.13	43.15 ± 8.79	49.22 ± 8.72	6.45 ± 0.2 ^{e}	10.62 ± 0.47	36.18 ± 1.41 ^{ee}	29.4 ± 0.58 ^{ee}	15.82 ± 0.72	1.61 ± 0.12
3	19.53 ± 2.35	40.58 ± 4.5	48.98 ± 5.63	6.4 ± 0.72 ^{eefg}	9.82 ± 0.98	34.58 ± 3.46 ^{eeffgg}	28.4 ± 0.15	17 ± 1.36	1.57 ± 0.03 ^{d}
4	22.11 ± 3.92 ^{dd}	33.08 ± 8.56	58.74 ± 7.41	5.72 ± 0.32 ^{deeffgg}	9.28 ± 0.8	33.11 ± 3.39 ^{deeffgg}	27.98 ± 0.51	17.36 ± 1.28	1.61 ± 0.11
5	24.32 ± 5.58 ^{g}	46.04 ± 15.83	47.4 ± 15.05	6.51 ± 0.32 ^{efg}	10.86 ± 0.54	36.94 ± 1.03 ^{ee}	29.42 ± 1.06 $^{c\ ee}$	15.68 ± 1.09	1.58 ± 0.11 ^{d}
6	18.95 ± 3.24	36.44 ± 8.55	57.1 ± 8.63	6.76 ± 0.21	11.12 ± 0.98 ^{c}	38.36 ± 3.09 ^{ee}	28.94 ± 0.45 ^{ee}	15.4 ± 0.54 ^{a}	1.44 ± 0.05
7	12.8 ± 2.76	27.76 ± 4.46 ^{c}	66.38 ± 4.35 ^{abc}	6.62 ± 0.64 ^{e}	11.16 ± 0.65 ^{c}	38.9 ± 2.43 ^{e}	28.66 ± 0.48	15.26 ± 0.54 ^{ac}	1.46 ± 0.06
8	20 ± 3.99	26.66 ± 4.97 ^{ac}	65.22 ± 4.53 ^{ac}	7.65 ± 0.54	12.12 ± 0.71 ^{abbcc}	44.3 ± 2.77	27.4 ± 0.46	15.42 ± 0.51 ^{a}	1.39 ± 0.03
9	22.76 ± 4.00 ^{d}	35.24 ± 6.74	56.72 ± 7.87	7.56 ± 0.32	12.18 ± 0.74 $^{aa\ bb\ cc}$	42.28 ± 1.68	28.8 ± 0.82 ^{e}	15.22 ± 0.75 ^{ac}	1.51 ± 0.04
10	15.2 ± 2.04	27.14 ± 5.43^{ac}	64.94 ± 6.51^{ac}	7.56 ± 0.36	12.28 ± 1.13 ^{aabbcc}	42.4 ± 3.65	29 ± 0.34	15.34 ± 0.66 ^{a}	1.56 ± 0.06

Key: C – control group; WBC - White Blood Cells; NEUT - Neutrophils; LYMPH - Lymphocytes; RBC - Red Blood Cells; HGB - Haemoglobin; HCT - Haematocrit; MCHC - Mean Corpuscular Haemoglobin Concentrations; RDW - Red Blood Cell Distribution Width; HDW - Haemoglobin Distribution Width. Statistical symbols: ^{a} – relative to date 1; ^{b} – relative to date 3; ^{c} – relative to date 5; ^{d} – relative to date 7; ^{e} – relative to date 8; ^{f} – relative to date 9; ^{g} – relative to date 10. Statistically significant differences: ^{a},^{b},^{c},^{d},^{e},^{f} and ^{g} at $P \leq 0.05$; ^{aa},^{bb},^{cc},^{dd},^{ee},^{ff} and ^{gg} at $P \leq 0.01$.
